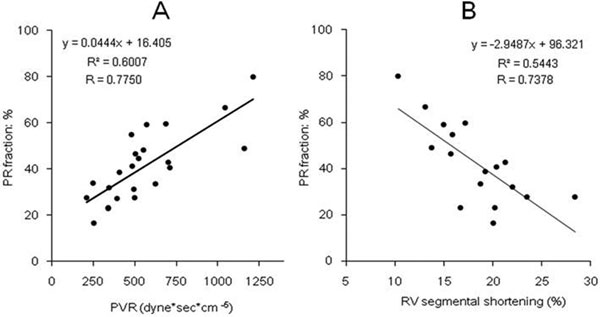# Impact of nitric oxide on pulmonary regurgitation and cardiac function in the acute stage after right ventricular outflow surgery

**DOI:** 10.1186/cc13370

**Published:** 2014-03-17

**Authors:** Y Ko, K Morita, R Nagahori, T Abe, K Hashimoto

**Affiliations:** 1 Jikei University School of Medicine, Tokyo, Japan

## Introduction

Pulmonary regurgitation (PR) that develops after right ventricular (RV) outflow reconstruction including the Rastelli and Norwood procedure may often result in serious cardiac events early after surgery. We hypothesized that PR may be associated with pulmonary vascular resistance (PVR) and RV contraction. Accordingly, we assessed the impact of PVR on PR and RV function using a swine model.

## Methods

Eight pigs (14 ± 2 kg) underwent total resection of the pulmonary valve cusps under cardiopulmonary bypass (PR group). This was compared with a control group (*n *= 6) that underwent only bypass. In both groups, the pulmonary regurgitant fraction (PRF) and cardiac output were measured by a pulsed Doppler flow meter, and the percent segmental shortening of RV (%RVSS) and RV end-diastolic dimension (RVDd) were measured by sonomicrometry. We also performed dobutamine stress evaluation as well as changing the PVR by carbon dioxide (PaCO_2_) and inhaled nitric oxide (NO).

## Results

All bypass time was 18 ± 3 minutes. In the PR group, the PRF was 40 ± 4% and the RVDd was 53 ± 9 mm* (vs. control 34 ± 6 mm). *P < 0.05. A significant reduction in the %RVSS (18 ± 1%* vs. control 22 ± 1%) and the cardiac output (2.1 ± 0.2 l/minute* vs. control 2.5 ± 0.3 l/minute) were observed. The PRFs were 60 ± 5% (PaCO_2 _>80 mmHg), 37 ± 2% (PaCO_2 _<20 mmHg), 24 ± 2% (NO 20 ppm; PaCO_2 _40 mmHg), and were positively correlated with the PVR (Figure 1A). During the dobutamine stress, the %RVSS was increased (baseline 18 ± 1%, 5γ 21 ± 2%, 10γ 26 ± 3%), and was negatively correlated with the PRFs (Figure 1B).

## Conclusion

These results indicated that massive PR resulted in marked deterioration of RV performance; however, low PVR and high RV contractility may contribute to reduce the severity of PR and improve cardiac function. Nitric oxide may be a useful treatment modality the same as catecholamine during the acute stage after RV outflow surgery with PR.

**Figure 1:**